# Time-Course of Knee Muscle Strength Recovery at 3, 6, and 12 Months Postoperatively After Open Wedge High Tibial Osteotomy: Differential Recovery Patterns of Maximal Power and Muscle Endurance

**DOI:** 10.3390/jcm15031214

**Published:** 2026-02-04

**Authors:** O-Sung Lee, Seung Ik Cho, Hyuntae Lee, Joon Kyu Lee

**Affiliations:** 1 Department of Orthopedic Surgery, Eulji University School of Medicine, Uijeongbu-si 11759, Republic of Korea; xixzeus@naver.com; 2 Department of Orthopaedic Surgery, Konkuk University Medical Center, Seoul 05030, Republic of Korea; bestjjo@hanmail.net (S.I.C.); 2cabca@naver.com (H.L.)

**Keywords:** osteotomy, muscle strength, muscle weakness, rehabilitation

## Abstract

**Objectives:** This study aimed to evaluate longitudinal changes in knee muscle strength following open wedge high tibial osteotomy (OWHTO) for medial compartment knee osteoarthritis with varus deformity, with particular emphasis on differences between the operated and non-operated knees. **Methods:** This retrospective study included 78 patients who underwent OWHTO. All patients followed a standardized rehabilitation protocol consisting of protected weight-bearing for six weeks, followed by closed kinetic chain exercises and subsequent open kinetic chain exercises from three months postoperatively. Isokinetic knee extension and flexion strength were assessed preoperatively and at 3, 6, and 12 months postoperatively using a Biodex System IV dynamometer at angular velocities of 60°/s and 180°/s. Absolute muscle strength values and inter-limb strength deficits were analyzed. Statistical analyses were performed using the Shapiro–Wilk, Friedman, and Wilcoxon signed-rank tests, with significance set at *p* ≤ 0.05. **Results:** At 60°/s, knee extensor and flexor strength deficits significantly increased after surgery, peaking at three months postoperatively, and gradually improved; however, deficits remained significantly greater than preoperative values at one year (*p* < 0.05). Similar trends were observed at 180°/s, although they did not reach statistical significance. These deficits were primarily attributable to reduced muscle strength in the operated knee, while strength in the non-operated knee remained unchanged throughout follow-up. **Conclusions:** Knee muscle strength in the operated limb markedly declined during the first three months following OWHTO, particularly in maximal power, and generally required more than six months to recover toward preoperative levels. These findings emphasize the importance of targeted postoperative rehabilitation strategies focusing on early muscle power recovery after OWHTO.

## 1. Introduction

Osteoarthritis (OA) of the knee is a major global health burden and a leading cause of disability worldwide. Approximately 27% of patients with knee OA present with isolated medial tibiofemoral compartment involvement. In these patients, particularly younger and physically active individuals, open wedge high tibial osteotomy (OWHTO) is a well-established surgical option that corrects varus malalignment, redistributes mechanical load away from the medial compartment, relieves pain, and delays the need for total knee arthroplasty (TKA) [1,2,3,4,5,6].

Despite its clinical effectiveness, OWHTO requires a postoperative period of protected weight-bearing, typically lasting around six weeks, to allow for bone healing and mechanical stability [7,8]. This period of reduced loading, compounded by arthrogenic muscle inhibition (a presynaptic reflexive inhibition of musculature surrounding an injured or postoperative joint) is associated with a rapid decline in periarticular muscle strength, particularly in the quadriceps and hamstrings [9]. Previous studies have reported quadriceps strength reductions of up to 60% within the first month after surgery, leading to impaired mobility, reduced balance, limitations in daily activities, and an increased risk of falls [10,11,12].

In addition, weakened periarticular muscles may compromise knee joint stability and protection, potentially accelerating cartilage degeneration and negatively affecting long-term surgical outcomes [13,14]. Although postoperative rehabilitation is routinely implemented after OWHTO, the detailed time course of knee muscle strength recovery remains insufficiently defined. A clearer understanding of these longitudinal changes is essential for optimizing rehabilitation strategies and improving functional recovery [15,16].

Therefore, the purpose of this study was to investigate time-dependent changes in knee extensor and flexor muscle strength following OWHTO. Especially, we aimed to: (1) evaluate the differential recovery trajectories between maximal muscle power (tested at 60°/s) and muscle endurance (tested at 180°/s); (2) quantify the magnitude of strength deficits by comparing the operated limb with the non-operated contralateral limb as an internal control. By addressing these time-course of knee muscle strength recovery, this study aims to identify the critical period of maximal strength loss and subsequent recovery plateaus and provide a more comprehensive clinical framework for optimizing rehabilitation protocols after OWHTO.

## 2. Materials and Methods

### 2.1. Study Design and Participants

Between July 2016 and July 2020, we retrospectively reviewed patients who underwent medial OWHTO for medial compartment knee osteoarthritis and varus deformity [1,2]. Only patients who completed both clinical and radiological follow-ups at one year postoperatively were included in the final analysis. The study was approved by the institutional review board (KUMC 2022-07-069).

### 2.2. Postoperative Rehabilitation

Postoperatively, knee range of motion exercises were initiated gradually following drain removal on the first postoperative day. Patients were instructed to maintain protected weight-bearing for six weeks. Regarding the exercise protocol, open kinetic chain exercises, including isometric quadriceps sets and progressive resisted knee extensions, were permitted at six weeks. These exercises were typically performed in 3 sets of 10–15 repetitions, with intensity adjusted based on the patient’s pain level and tolerance. This was followed by the introduction of closed kinetic chain exercises, such as mini-squats (0–45°) and leg presses with graduated resistance at three months postoperatively [5,6]. All exercises were progressed by increasing resistance or repetitions as the patient demonstrated improved muscle control and decreased joint irritability.

### 2.3. Isokinetic Muscle Strength Assessment

Knee muscle strength was evaluated preoperatively and at 3, 6, and 12 months postoperatively using a Biodex System 4 dynamometer (Biodex Medical Systems Inc., Shirley, NY, USA) [6]. Isokinetic knee extension and flexion strengths were measured for both the operated and non-operated knees at angular velocities of 60°/s (maximal power) and 180°/s (muscle endurance) [6]. During the assessment, the trunk, pelvis, and thigh were secured with straps to minimize compensatory movements, and the range of motion was set according to each patient’s tolerance. Gravity correction was performed for each limb to eliminate the effect of leg weight on torque measurements. Absolute muscle strength values (Peak Torque, N·m) were recorded, and strength deficits (%) were calculated as follows to quantify the inter-limb asymmetry at each time point to evaluate functional recovery [17].

### 2.4. Statistical Analysis

Statistical analyses were performed using SPSS version 21.0 (IBM Corp., Armonk, NY, USA). Data normality was assessed using the Shapiro–Wilk test. Differences across time points were analyzed using the Friedman test, followed by the Wilcoxon signed-rank test for post hoc comparisons. Statistical significance was defined as *p* < 0.05.

### 2.5. AI-Assisted Writing and Editing

During the preparation of this manuscript, the authors used Gemini 3 Flash (Google, Mountain View, CA, USA) solely for the purpose of English language editing and grammatical refinement. The AI tool was not used for data collection, analysis, or the generation of original scientific content. The authors reviewed and edited the output as needed and take full responsibility for the content of the published article.

## 3. Results

### 3.1. Patients’ Characteristic

A total of 97 consecutive patients underwent OWHTO during the study period. Patients were included if they had isolated medial compartment osteoarthritis (Kellgren-Lawrence grade 2 or 3) and a varus deformity. Exclusion criteria were pre-defined as follows: (1) inflammatory arthritis such as rheumatoid arthritis, (2) history of previous major knee surgery or fracture on the affected side, and (3) severe patellofemoral or lateral compartment OA. Nineteen patients were excluded based on the following specific criteria: (1) inadequate follow-up, defined as missing more than one scheduled isokinetic muscle strength test at 3, 6, or 12 months; (2) postoperative complications that hindered standardized rehabilitation, such as deep surgical site infection, hardware failure, or symptomatic nonunion; (3) bilateral knee surgery performed within a one-year interval to avoid confounding the strength of the non-operated limb; and (4) incomplete clinical or radiographic data. The final study cohort consisted of 78 patients (25 males, 32.1%, and 53 females, 67.9%) with a mean age of 56.8 ± 8.1 years and a mean body mass index of 27.3 ± 4.0 kg/m^2^. Surgical indications included active patients younger than 70 years of age, including those with mild patellofemoral arthritis.

### 3.2. Extensor and Flexor Strength Deficits at 60°/s (Table 1)

At an angular velocity of 60°/s, both knee extensor and flexor strength deficits of the operated knee significantly increased after surgery, peaking at three months postoperatively. Extensor deficits increased from 30.37% ± 16.59% preoperatively to 52.77% ± 17.54% at three months, while flexor deficits increased from 20.73% ± 20.50% to 39.06% ± 18.56%. Following this peak, strength deficits gradually improved over time. At six months, extensor and flexor deficits decreased to 40.65% ± 16.99% and 21.89% ± 17.58%, respectively, and further improved to 26.17% ± 19.12% and 15.09% ± 18.15% at one year. All inter-time point comparisons showed statistically significant differences (*p* < 0.05). Notably, at one year postoperatively, mean extensor and flexor deficits were numerically lower than preoperative values, indicating recovery beyond the initial baseline.

**Table 1:** Muscle strength deficits (operated knee compared to non-operated knee) according to the time point evaluated with a Biodex system IV dynamometer (60°/s).

	Extensor, %	Flexor, %
Preoperative	30.37 ± 16.59	20.73 ± 20.50
3 months postoperative	52.77 ± 17.54	39.06 ± 18.56
6 months postoperative	40.65 ± 16.99	21.89 ± 17.58
1 year postoperative	26.17 ± 19.12	15.09 ± 18.15

The values are given as the mean and the standard deviation.

### 3.3. Extensor and Flexor Strength Deficits at 180°/s (Table 2)

At 180°/s, a similar temporal pattern was observed. Extensor deficits increased from 23.53% ± 16.70% preoperatively to 42.38% ± 14.37% at three months and subsequently decreased to 31.01% ± 12.51% at six months and 20.32% ± 13.30% at one year. Flexor deficits increased from 13.37% ± 20.11% to 23.85% ± 16.36% at three months, followed by improvement to 17.20% ± 14.74% at six months and 9.40% ± 14.79% at one year. Although comparisons between preoperative and three-month postoperative time point were statistically significant in extensor and flexor strength (*p* < 0.05), longer-term comparisons between preoperative and later postoperative time points did not consistently reach statistical significance.

**Table 2:** Muscle strength deficits (operated knee compared to non-operated knee) according to the time point evaluated with a Biodex system IV dynamometer (180°/s).

	Extensor, %	Flexor, %
Preoperative	23.53 ± 16.70	13.37 ± 20.11
3 months postoperative	42.38 ± 14.37	23.85 ± 16.36
6 months postoperative	31.01 ± 12.51	17.20 ± 14.74
1 year postoperative	20.32 ± 13.30	9.40 ± 14.79

The values are given as the mean and the standard deviation.

### 3.4. Actual Muscle Strength at 60°/s (Table 3 and Table 4)

At 60°/s, the actual extensor strength of the operated knee significantly decreased from 74.65 ± 31.40 N·m preoperatively to 46.72 ± 21.09 N·m at three months postoperatively. Strength progressively recovered to 62.96 ± 28.62 N·m at six months and nearly returned to preoperative levels by one year (74.38 ± 27.81 N·m). Significant differences were observed in all inter-time point comparisons (*p* < 0.05), except for the comparison between preoperative and one-year postoperative strength in the operated knee (*p* = 0.092).

**Table 3:** Actual extensor muscle strengths of the operated and the non-operated knees according to the time point evaluated with a Biodex system IV dynamometer (60°/s).

	Operated Knee, N∙m	Non-Operated Knee, N∙m
Preoperative	74.65 ± 31.40	108.22 ± 38.32
3 months postoperative	46.72 ± 21.09	103.65 ± 40.69
6 months postoperative	62.96 ± 28.62	107.87 ± 38.67
1 year postoperative	74.38 ± 27.81	104.83 ± 38.43

The values are given as the mean and the standard deviation.

Similarly, flexor strength of the operated knee decreased from 41.37 ± 18.14 N·m preoperatively to 31.05 ± 15.09 N·m at three months, followed by recovery to 40.71 ± 18.88 N·m at six months and 44.73 ± 16.86 N·m at one year. Statistically significant differences were observed across all time points (*p* < 0.05), with the exception of the comparison between preoperative and 6-month postoperative strength in the operated knee (*p* = 0.084), and between preoperative and 3-month postoperative strength in the non-operated knee (*p* = 0.074).

**Table 4:** Actual flexor muscle strengths of the operated and the non-operated knees according to the time point evaluated with a Biodex system IV dynamometer (60°/s).

	Operated Knee, N∙m	Non-Operated Knee, N∙m
Preoperative	41.37 ± 18.14	52.33 ± 20.85
3 months postoperative	31.05 ± 15.09	51.54 ± 21.81
6 months postoperative	40.71 ± 18.88	51.86 ± 20.39
1 year postoperative	44.73 ± 16.86	53.20 ± 19.54

The values are given as the mean and the standard deviation.

### 3.5. Actual Muscle Strength at 180°/s (Table 5 and Table 6)

At 180°/s, extensor strength of the operated knee declined from 54.59 ± 20.30 N·m preoperatively to 41.18 ± 17.97 N·m at three months and subsequently recovered to 49.09 ± 18.25 N·m at six months and 54.27 ± 21.16 N·m at one year. Although most comparisons demonstrated significant differences (*p* < 0.05), the difference between preoperative and one-year values was not statistically significant. Statistically significant differences were observed across all time points (*p* < 0.05), with the exception of the comparison between preoperative and 1-year postoperative strength in the operated knee (*p* = 0.174).

**Table 5:** Actual extensor muscle strengths of the operated and the non-operated knees according to the time point evaluated with a Biodex system IV dynamometer (180°/s).

	Operated Knee, N∙m	Non-Operated Knee, N∙m
Preoperative	54.59 ± 20.30	72.47 ± 24.63
3 months postoperative	41.18 ± 17.97	73.82 ± 32.48
6 months postoperative	49.09 ± 18.25	71.35 ± 23.80
1 year postoperative	54.27 ± 21.16	68.85 ± 24.88

The values are given as the mean and the standard deviation.

Flexor strength at 180°/s followed a similar pattern, decreasing from 33.33 ± 16.36 N·m preoperatively to 29.90 ± 15.16 N·m at three months and improving to 35.21 ± 16.49 N·m at six months and 35.70 ± 16.11 N·m at one year. Strength values of the non-operated knee remained stable throughout follow-up, with no statistically significant changes observed. Statistically significant differences were observed across all time points (*p* < 0.05), with the exception of the comparisons between preoperative and 6-month postoperative strength (*p* = 0.079), as well as between 6-month and 1-year postoperative strength in the operated knee (*p* = 0.189).

**Table 6:** Actual flexor muscle strengths of the operated and the non-operated knees according to the time point evaluated with a Biodex system IV dynamometer (180°/s).

	Operated Knee, N∙m	Non-Operated Knee, N∙m
Preoperative	33.33 ± 16.36	38.24 ± 15.68
3 months postoperative	29.90 ± 15.16	39.54 ± 18.64
6 months postoperative	35.21 ± 16.49	41.67 ± 16.60
1 year postoperative	35.70 ± 16.11	39.55 ± 16.34

The values are given as the mean and the standard deviation.

## 4. Discussion

This study provides a longitudinal analysis of knee muscle strength recovery following OWHTO, revealing significant time-dependent changes in the operated limb [1]. The most notable finding is the acute and substantial decrease in both knee extensor and flexor strength in the operated knee, peaking at 3 months post-surgery, particularly evident at the 60°/s isokinetic testing speed which measures maximal power [10,11]. While strength gradually improved thereafter, it generally took over 6 months to recover to preoperative levels with some residual deficits persisting at one year, particularly for maximal power [4,17]. The differential recovery patterns between 60°/s (power) and 180°/s (endurance) protocols highlight that maximal power is more severely affected and requires more targeted attention during rehabilitation.

The pronounced decline in knee muscle strength observed at three months postoperatively has important clinical implications. This time point closely follows the initial six-week period of protected weight-bearing, which is essential for bone healing and mechanical stability after OWHTO [7,8]. However, this necessary reduction in loading inevitably contributes to muscle disuse atrophy. In addition, postoperative pain and joint effusion can induce arthrogenic muscle inhibition, a well-recognized neural mechanism that limits voluntary muscle activation, particularly of the quadriceps [12]. The combined effects of mechanical unloading and neural inhibition likely explain the magnitude of strength loss observed in the early postoperative period.

The differential recovery patterns between maximal power and muscle endurance observed in this study provide further insight into postoperative functional impairment after OWHTO. Strength deficits measured at 60°/s were more pronounced and persisted longer than those measured at 180°/s, suggesting that high-force, rapid muscle contractions are more vulnerable to surgical insult and immobilization [10,11]. This finding is clinically relevant because maximal muscle power is a critical determinant of functional performance in activities such as stair climbing, rising from a seated position, gait acceleration, and fall prevention [17]. Therefore, recovery of muscle endurance alone may not be sufficient to restore optimal knee function if power deficits persist.

In addition, the differential recovery patterns observed between extensor and flexor muscles, as well as between maximal power (60°/s) and endurance-oriented (180°/s) protocols, warrant further consideration. Although both muscle groups demonstrated a similar temporal trend of early postoperative decline followed by gradual recovery, extensor strength—particularly at lower angular velocities—exhibited a more pronounced and prolonged deficit. This finding is clinically relevant, as quadriceps power plays a critical role in functional activities such as stair negotiation, sit-to-stand transitions, and dynamic knee stability [10,11,12]. Previous studies have suggested that altered knee biomechanics and unloading of the medial compartment after OWHTO may transiently reduce quadriceps activation efficiency, potentially delaying power recovery despite structural realignment [13,14]. Therefore, the persistence of extensor power deficits, even when endurance measures appear to normalize, may represent a subclinical limitation that is not adequately captured by conventional strength assessments alone [15,16].

An important finding of this study is the relative stability of muscle strength in the non-operated knee throughout the postoperative period. This observation supports the interpretation that the observed strength deficits were specific to the operated limb rather than the result of systemic deconditioning or aging-related changes [6]. The preserved strength of the contralateral limb suggests a potential role for contralateral or cross-education training strategies during the protected weight-bearing phase. Eccentric or high-intensity training of the non-operated limb may induce neural adaptations that facilitate strength preservation or recovery in the operated knee [15].

OWHTO is commonly performed in younger and more active patients with medial compartment knee osteoarthritis, for whom long-term functional performance and activity participation are critical goals [1,2,3]. Unlike patients undergoing total knee arthroplasty, this population often seeks to return to high-demand occupational or recreational activities. Our findings emphasize that early postoperative muscle weakness, particularly loss of maximal power, may represent a key barrier to achieving these goals. Accordingly, rehabilitation protocols should aim not only to restore basic strength but also to incorporate progressive loading and power-oriented exercises once adequate bone healing has been achieved [4,5]. This study has several limitations. First, its retrospective design limited the availability of patient-reported outcome measures, preventing direct assessment of the relationship between objective muscle strength recovery and subjective functional improvement. Second, detailed radiologic evaluation of bone union was not available, precluding analysis of the association between the timing of bony healing and muscle strength recovery [7,16]. Third, the follow-up period was limited to one year, and longer-term differences in muscle strength between the operated and non-operated knees could not be evaluated [1,2]. Finally, although isokinetic testing provides reliable and objective measurements, it may not fully reflect functional performance during daily or sport-specific activities. This study did not incorporate standardized functional assessments, such as the timed up-and-go or stair climb test, which could provide further insight into the clinical relevance of the observed power deficits. Furthermore, future research utilizing advanced gait rehabilitation analysis—including wearable sensors or cyber-physical systems—is warranted to objectively evaluate how these muscle strength changes correlate with dynamic gait kinematics and real-world functional recovery [18]. Combining isokinetic data with such sophisticated functional and biomechanical assessments will offer a more comprehensive understanding of postoperative rehabilitation outcomes after OWHTO.

## 5. Conclusions

Knee muscle strength in the operated limb significantly decreased during the initial 3 months after OWHTO, particularly maximal power, and generally required over 6 months to recover to preoperative levels. The non-operated knee’s strength remained stable throughout the observation period. These findings highlight the need for targeted rehabilitation focusing on power recovery and potentially incorporating strategies during the early postoperative phase. Based on these findings, postoperative rehabilitation after OWHTO should not be limited to gradual strength restoration but should also emphasize the recovery of maximal muscle power once sufficient bone healing is achieved. Early identification of prolonged strength deficits may help clinicians individualize rehabilitation intensity and timing to optimize functional recovery.

## Data Availability

The data presented in this study are available from the corresponding author upon reasonable request due to ethical and privacy restrictions.
